# CaO–SiO_2_–P_2_O_5_–B_2_O_3_-Based Bioactive Glass (BGS-7) Macrobeads Incorporated in Hydrogels Aid Bone Regeneration: Evaluation in Rabbit Calvarial and Femoral Defect Models

**DOI:** 10.3390/ma19020309

**Published:** 2026-01-12

**Authors:** Wonseok Choi, Seonghyun Kang, Eliel Nham, Seung-hyo Go, Do-yeon Lee, Baek-Hyun Kim, Jong-Keon Oh

**Affiliations:** 1 Department of Orthopedic Surgery, Korea University Guro Hospital, Korea University College of Medicine, Seoul 08308, Republic of Korea; wscool@korea.ac.kr (W.C.); ss7186@korea.ac.kr (S.K.); 2 Division of Infectious Diseases, Department of Medicine, Korea University Guro Hospital, Seoul 08308, Republic of Korea; e.nham@kumc.or.kr; 3 Research Center, CGBio Co., Ltd., CG Building, Seoul 04349, Republic of Korea; shgo98@cgbio.co.kr (S.-h.G.); idoyeoni@cgbio.co.kr (D.-y.L.); bhkim82@cgbio.co.kr (B.-H.K.)

**Keywords:** bone regeneration, bioactive glass macrobeads, hydrogel formulations, bone graft

## Abstract

Bone graft substitutes are extensively investigated for addressing critical-size bone defects; however, their efficacy is limited by inadequate bone regeneration and subpar handling properties. Herein, we compared the bone regenerative capacity of CaO–SiO_2_–P_2_O_5_–B_2_O_3_-based bioactive glass (BGS-7) macrobeads with that of β-tricalcium phosphate (β-TCP) beads and evaluated their performance when incorporated into hydrogels to improve their handling properties. BGS-7 macrobeads were fabricated via alginate crosslinking and heat treatment, and their physicochemical properties and microstructures were characterized. In a rabbit calvarial defect model, BGS-7 macrobeads, heat-treated at 600 and 800 °C, exhibited superior bone bridging and degradation than size-matched β-TCP macrobeads. To further evaluate their regenerative potential, critical-size defects (6 mm diameter × 10 mm depth) were created in the rabbit femoral condyle. To enhance clinical applicability, BGS-7 beads were incorporated into cellulose-based hydrogels and implanted into the defects. Radiographic and histomorphometric analyses demonstrated that bone formation and stable fixation achieved with hydrogel formulations containing BGS-7 microbeads and Laponite were more pronounced than those with BGS-7 beads alone. The findings suggest that BGS-7 macrobeads, particularly when combined with microbead- and Laponite-containing hydrogels, represent a promising bone graft substitute with improved regenerative and handling properties compared with using BGS-7 beads alone.

## 1. Introduction

Infected bone defects present a major clinical challenge in orthopedic and reconstructive surgeries, typically arising from trauma, surgical complications, or implant-related infections. These defects present a dual therapeutic challenge, namely, the complete eradication of pathogenic microorganisms and restoration of bone continuity and function [1,2,3,4,5,6,7,8,9,10,11,12].

The management of infected bone defects places a substantial burden on both patients and healthcare systems and is characterized by prolonged treatment periods, multiple surgical interventions, and high failure rates that can lead to persistent disability and reduced quality of life [7,8]. The complexity is further compounded by the need to address both the infectious process and underlying bone loss simultaneously, creating an urgent clinical need for innovative biomaterials that can effectively combat infection while promoting bone regeneration and healing.

The current clinical management of infected bone defects follows a well-established protocol involving surgical debridement, systemic antibiotic therapy, local antibiotic delivery (commonly through polymethyl methacrylate [PMMA] beads), and bone grafting procedures, often performed in multiple-stage operations [2,5,6,11]. However, conventional approaches have several limitations that compromise treatment efficacy and patient outcomes. Systemic antibiotics frequently demonstrate poor penetration into bone tissue owing to limited vascular supply, particularly in infected or necrotic areas, resulting in suboptimal local drug concentrations [9,10,11]. Non-biodegradable carriers, such as PMMA beads, require additional surgical procedures for their removal, and this increases patient morbidity and healthcare costs. Most critically, current treatment modalities lack materials that can simultaneously deliver therapeutic agents and actively promote bone healing, necessitating separate interventions for infection control and bone regeneration. These limitations have sparked growing interest in the development of biodegradable multifunctional biomaterials capable of providing sustained local drug delivery while serving as osteoconductive scaffolds for bone repair [1,10,11,12].

Bioactive glass, originally developed by Hench in 1969, is a silica-based material that forms an apatite layer on its surface upon exposure to physiological fluids, thereby establishing a direct bond with bones [13]. Owing to its osteoconductive properties, capacity to stimulate bone regeneration, and controllable degradation behavior, bioactive glass has been extensively investigated as a substitute for bone grafts [13,14,15,16,17,18,19,20,21,22].

Despite promising in vitro results demonstrating the potential of bioactive glass as both an osteoconductive scaffold and a drug carrier, in vivo data remain limited, particularly in standardized defect models that can provide reliable preclinical evidence. Previous studies have reported encouraging results with bioactive glass in various animal models, such as rat calvarial defect and rabbit radius ostectomy models, and in periodontal applications in dogs [23,24,25]. However, obtaining comprehensive biocompatibility and bone regenerative performance data in controlled experimental environments is essential before introducing the additional complexity of infection models. The systematic evaluation of bioactive glass scaffolds in critical-size bone defects represents an essential foundational step, as demonstrated by studies showing variable healing responses at different anatomical locations and defect sizes [26,27,28].

BGS-7, a CaO–SiO_2_–P_2_O_5_–B_2_O_3_-based bioactive glass, developed by CGBio (Seoul, Republic of Korea), demonstrates excellent osteointegration through rapid surface apatite formation [22]. In the present study, we aimed to evaluate the bone regeneration capability of an implantable BGS-7 bioactive glass-based device using a rabbit model of critical-size bone defects, providing essential preclinical data that can promote its application in infected bone defects and advancing the development of next-generation therapeutic strategies for complex bone reconstruction.

## 2. Materials and Methods

### 2.1. Preparation of Materials

#### 2.1.1. Fabrication of Macrobeads and Microbeads

The experimental compositions are listed in Table 1. Bioactive glass macrobeads were fabricated using BGS-7 glass powder. The BGS-7 glass powder was synthesized in-house following the exact composition and fabrication procedure previously reported by Lee et al. [22]. Sodium alginate (13035S1201; Junsei Chemical, Tokyo, Japan) was used as a spherical-forming agent and dissolved in 1 L of deionized water at a concentration of 16.5 g·L^−1^. A designated binder—either poly(vinyl alcohol) (PVA; PVA205, Kuraray, Osaka, Japan) or polyethylene glycol (PEG; PEG200, Samchun Chemical, Jincheon, Republic of Korea)—was added at 1.0 wt% (0.3 g) relative to the BGS-7 powder (proprietary concentration). The solution was homogenized using a high-shear mixer (TM001; Revorox, Seoul, Republic of Korea) at 3000 rpm for more than 30 min at room temperature to ensure uniform mixing. Subsequently, 33 g of BGS-7 powder was added, and the mixture was homogenized to achieve a uniform dispersion.

Crosslinking was performed using a 1 g·L^−1^ CaCl_2_ solution. The precursor suspension was introduced into the cross-linking solution using an encapsulator (B-390; Buchi Labortechnik, Flawil, Switzerland), which generated droplets at 10–100 Hz according to the machine settings. Needles with internal diameters ranging from 100 to 1000 μm were used for droplet formation, resulting in the production of alginate-cross-linked BGS-7 macrobeads.

The crosslinked macrobeads were dried and heat-treated in a muffle furnace at a heating rate of 5 °C/min at 600, 700, and 800 °C under ambient air to eliminate alginate and then furnace-cooled to room temperature (~25 °C). The final macrobeads were sieved to a particle size range of 650–800 µm and sterilized using gamma irradiation at 30 kGy (Greenpiatech, Yeoju, Republic of Korea)

For comparison, β-tricalcium phosphate (β-TCP; Cerectron, Pyeongtaek, Republic of Korea) beads were prepared using the same procedure, followed by heat treatment at 1350 °C, sieved to obtain approximately 730 µm particles, and sterilized using gamma irradiation at 30 kGy.

BGS-7 microbeads were produced by adding 1% PVA binder to a slurry prepared at a 1:1 BGS-7 powder-to-water ratio, followed by the spray-drying method (Spray dryer; DJE-015R, Dongjin Eng., Ansan, Republic of Korea) at an inlet temperature of 180 °C and 8000 rpm. Microbeads smaller than 100 µm were then collected through sieving.

#### 2.1.2. Fabrication of Hydrogel

Hydrogels were prepared using hydroxypropyl methylcellulose (HPMC, H1136; Spectrum Chemical, New Brunswick, NJ, USA), carboxymethyl cellulose (CMC, C0045; Tokyo chemical industry, Tokyo, Japan), and polyacrylic acid (HK940; Hannong, Gyeongsan, Republic of Korea) as the main polymers, with BGS-7 microbeads and Laponite^®^ (Laponite XLG, BYK, Wesel, Germany) incorporated in some formulations. The hydrogels were mixed under controlled conditions using a vacuum paddle mixer (Twister Stand; Renfert GmbH, Hilzingen, Germany), filled into syringes, and sterilized using gamma irradiation. The hydrogel compositions are listed in Table 2. The exact ratios are proprietary, and hence, only the presence of each component is indicated.

#### 2.1.3. Microstructures of BGS-7 Macrobeads and Microbeads

Microstructures of the fabricated materials were evaluated using scanning electron microscopy (SEM; Helios 5 UC; Thermo Fisher Scientific, Waltham, MA, USA). The samples were sputter-coated with platinum, and imaging was performed at an accelerating voltage of 10 kV. The crystalline phases were analyzed using X-ray diffraction (XRD; Miniflex 600, Rigaku, Tokyo, Japan) over a 2 θ range of 20–40°, with a step size of 0.02° and a scanning speed of 5°/min (XRD; Miniflex 600; Rigaku, Tokyo, Japan). The specific surface area was measured by nitrogen adsorption using the Brunauer–Emmett–Teller (BET) method (Tristar II Plus, Micromeritics, Norcross, GA, USA).

### 2.2. Animal Preparations and Experimental Design

#### 2.2.1. Calvarial Defect Model

Ethical statement: All animal procedures were approved by the Institutional Animal Care and Use Committee (protocol code 202402-002, date of approval: 13 February 2024). Four healthy New Zealand white rabbits (5–6 months old, weighing 3.0–3.5 kg) were housed individually under standard laboratory conditions and provided a commercial diet and water ad libitum. Appropriate anesthesia, analgesia, and humane euthanasia were performed to minimize pain and distress.

General anesthesia was induced via intramuscular injection of tiletamine hydrochloride (5 mg/kg) and zolazepam hydrochloride (5 mg/kg) (Zoletil 50; Virbac, Carros, France) in combination with xylazine (5.0 mg/kg) (Rompum; Bayer, Leverkusen, Germany); the total injection volume was 1 mL. Local anesthesia was achieved through the infiltration of 2% lidocaine containing 1:100,000 epinephrine (Huons, Seongnam, Republic of Korea) into both calvarial sites.

**Figure 1:**
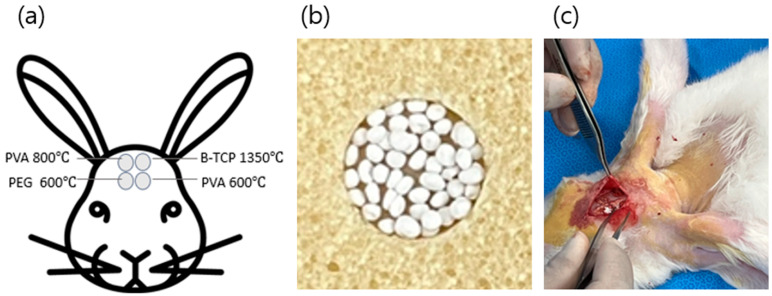
(**a**): location of the rabbit calvarial defect sites; (**b**): photograph of BGS-7 macrobeads and an application example; (**c**): intraoperative photograph of the actual implantation in rabbits.

A midline skin incision was made, and the periosteum was reflected to expose the calvarial surface. Four circular defects (6.0 mm in diameter) were created using a trephine bur under continuous saline irrigation until the inner cortical plate was completely removed. Sterile saline was continuously applied to minimize heat generation. Each defect was grafted under different beads conditions, as illustrated in Figure 1.

The surgical sites were disinfected with povidone–iodine solution and subjected to primary closure using polyglactin 4-0 sutures (Vicryl, Ethicon, Somerville, NJ, USA). Postoperative prophylaxis involved intramuscular administration of enrofloxacin (Biotril; Komipharm International, Jincheon, Republic of Korea).

To evaluate bone remodeling, two rabbits were sacrificed at 4 weeks postoperatively, and the remaining two were sacrificed at 8 weeks postoperatively via intravenous administration of potassium chloride (15 mg/kg).

#### 2.2.2. Femoral Condyle Defect Model

Ethical statement: All animal procedures were approved by the Institutional Animal Care and Use Committee (protocol code 202502-003, date of approval: 25 February 2025) Eight healthy New Zealand white rabbits (5–6 months old, 3.0–3.5 kg) were housed individually under standard laboratory conditions and provided a commercial diet and water ad libitum. Appropriate anesthesia, analgesia, and humane euthanasia were performed to minimize pain and distress. Four experimental groups were evaluated (*n* = 4/group).

**Figure 2:**
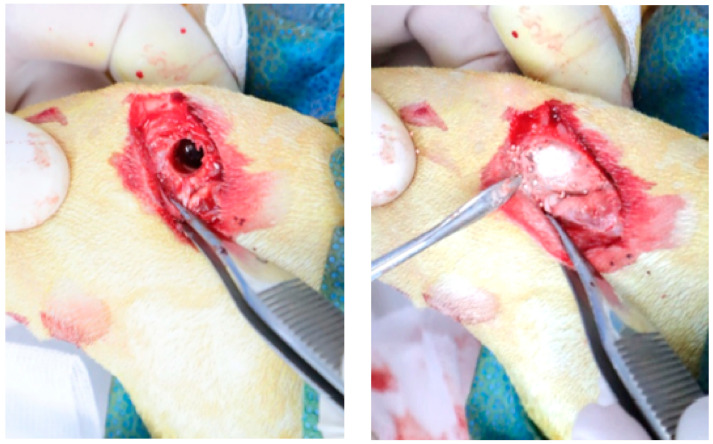
Intraoperative photograph of the rabbit femoral condyle defect implantation.

A 2 cm skin incision was made over the distal femoral condyle, and the muscles were bluntly dissected to expose the lateral femoral condyle. Critical-size defects (6 mm in diameter × 10 mm in depth, with a volume of approximately 0.3 cc) were created bilaterally in the distal epiphysis using a trephine bur under constant saline irrigation (Figure 2). Each defect was rinsed thrice with sterile saline. Hydrogels and BGS-7 beads were mixed intraoperatively using a connector system with repeated back-and-forth mixing to ensure homogeneity.

Bleeding was controlled, and the wound was closed in layers. Bacitracin ointment was applied topically, and the surgical sites were disinfected with povidone–iodine. Primary closure was performed with polyglactin 4-0 sutures. Postoperative medication was identical to that described for the calvarial defect model.

Animals were sacrificed at 8 weeks postoperatively to evaluate bone remodeling and fixation strength via intravenous administration of potassium chloride (15 mg/kg).

#### 2.2.3. Evaluation of Animal Study

Bone regeneration was evaluated using conventional radiography, micro-computed tomography (Micro-CT; Skyscan 1273, Kontich, Belgium), and histological analyses. Radiographs were obtained at 4 and 8 weeks with the animals under general isoflurane anesthesia to monitor new bone formation and bone union within the defects. After euthanasia, the specimens were dissected, soft tissues were removed, and Micro-CT analysis was performed using an X-ray source operated at 80 keV and 100 µA, with an isotropic resolution of 10 µm.

Samples were fixed in 4% paraformaldehyde, decalcified in 10% nitric acid, dehydrated, cleared, and embedded in paraffin. Serial 5-μm-thick sections were obtained in the sagittal plane parallel to the femoral axis, focusing on the defect-bridging region. Approximately 10 sections were obtained per specimen, deparaffinized, and stained with hematoxylin and eosin (H&E). A histological evaluation was performed under a light microscope to assess graft degradation and bone regeneration.

Statistical analyses were conducted using the Statistical Package for the Social Sciences (SPSS, version 13.0, IBM, Armonk, NY, USA). Radiographic and Micro-CT data were analyzed using two-way analysis of variance (ANOVA). Data are expressed as mean ± standard deviation (SD). Statistical significance was set at *p* < 0.05.

## 3. Results

### 3.1. Microstructure of BGS-7 Macrobeads and Microbeads

**Figure 3:**
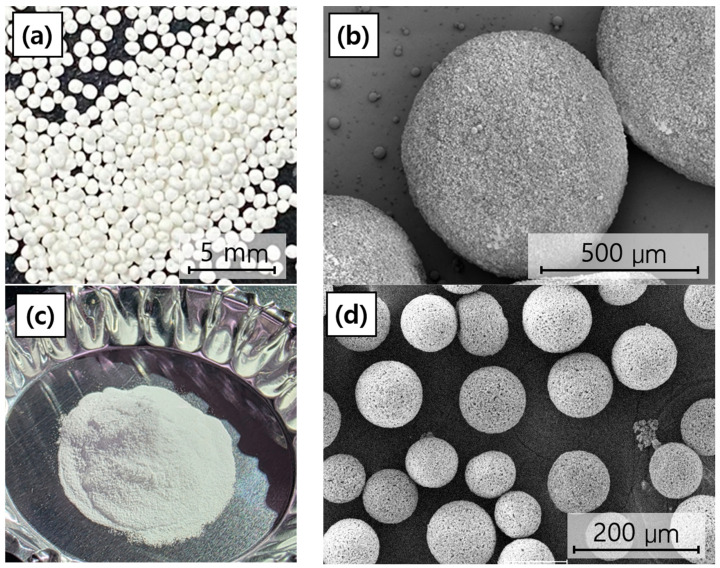
Overall morphology and SEM microstructure of BGS-7 macrobeads and microbeads: (**a**) overall morphology of BGS-7 macrobeads, (**b**) SEM microstructure of BGS-7 macrobeads at 150× magnification, (**c**) overall morphology of BGS-7 microbeads, and (**d**) SEM microstructure of BGS-7 microbeads at 300× magnification.

The morphology of the fabricated BGS-7 macrobeads and microbeads was first examined using SEM. As shown in Figure 3, both bead types exhibited an overall spherical shape with relatively uniform size distributions. The average particle size of the macrobeads was approximately 760 µm, whereas the microbeads exhibited an average particle size of approximately 80 µm. While the surfaces were not entirely smooth, they displayed a fine, granular, and porous texture, likely originating from the aggregation of smaller particles during the bead formation process. Such surface morphologies may be advantageous for providing an increased surface area, potentially enhancing ion exchange, dissolution, and subsequent bioactivity in physiological environments.

The microstructure of BGS-7 macrobeads was examined using both SEM and XRD to evaluate the morphological and crystallinity changes under different heat-treatment conditions.

**Figure 4:**
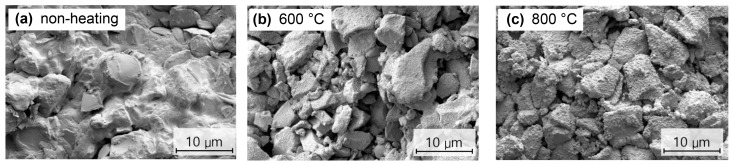
SEM images of BGS-7 macrobeads under different heat-treatment conditions: (**a**) non-heated, (**b**) at 600 °C, and (**c**) at 800 °C.

SEM images (Figure 4) revealed that the non-heated macrobeads had relatively smooth and irregular surfaces (Figure 4a). Heat treatment at 600 °C induced partial densification with small crystalline grains (Figure 4b), whereas at 800 °C, the beads displayed a compact structure with well-defined, interconnected grains, suggesting that higher heat-treatment temperatures promote grain growth and densification, potentially enhancing mechanical stability and influencing degradation behavior (Figure 4c).

**Figure 5:**
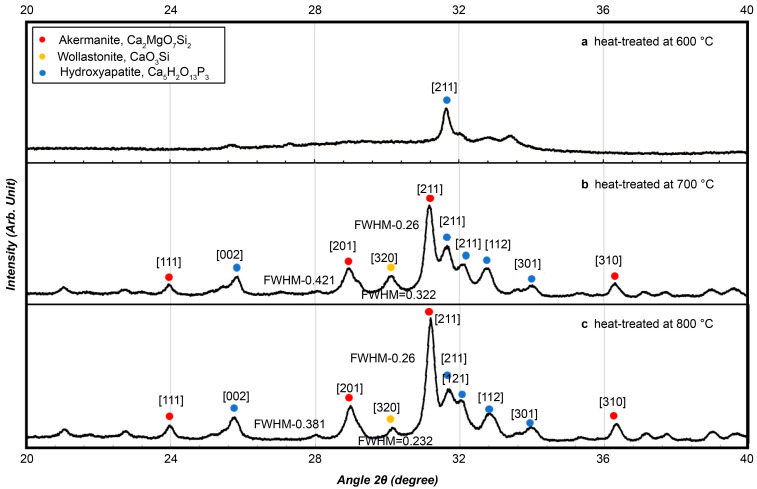
Crystallinity measurements were conducted for bioactive glass using X-ray diffraction at (**a**) 600 °C, (**b**) 700 °C, and (**c**) 800 °C.

The XRD analysis (Figure 5) showed that beads heat-treated at 600 °C retained a largely amorphous structure with a broad hydroxyapatite peak. At 700 °C, distinct crystalline phases including akermanite (Ca_2_MgO_7_Si_2_), wollastonite (CaSiO_3_), and hydroxyapatite (Ca_10_(PO_4_)_6_(OH)_2_) appeared, as indicated by sharp diffraction peaks. The peaks became more pronounced at 800 °C, confirming enhanced crystallinity and phase development at higher temperatures.

Although the 700 °C heat-treated samples exhibited distinct crystalline phases, preliminary evaluations revealed that phase transitions near this temperature were highly sensitive to small fluctuations in furnace conditions, making it difficult to obtain consistent material properties across batches. Therefore, only the 600 °C and 800 °C groups were selected for in vivo testing to ensure reproducibility and stable performance.

### 3.2. In Vivo Bone Formation Assessment in Rabbit Calvarial Defect Model

#### 3.2.1. Micro-CT Analysis

**Figure 6:**
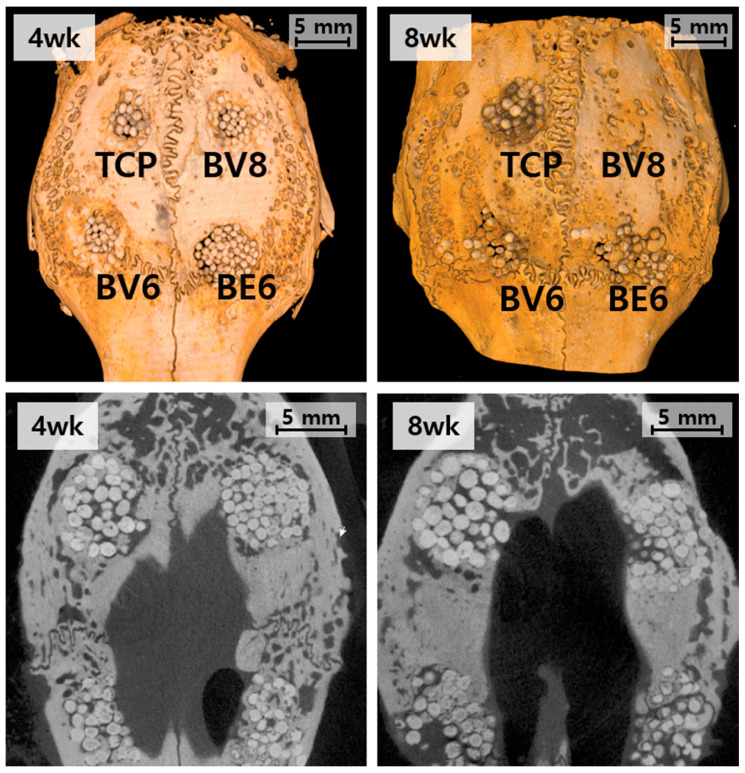
Micro-CT evaluation of the rabbit calvarial defect model at 4 and 8 weeks: top, three-dimensional reconstruction; bottom, cross-sectional images.

The Micro-CT analysis demonstrated a clear time-dependent progression of bone regeneration in all the experimental groups (Figure 6). The specific surface area (BET) of each graft material is presented in Table 3.

In the β-TCP control group (Group TCP), new bone formation remained limited. At 4 weeks, the bone was confined to the defect margins, with the implanted beads largely intact. By 8 weeks, partial resorption of the β-TCP beads was observed; however, substantial bone replacement was absent, and defect bridging was incomplete. Group BV6 (BGS-7, 600 °C, PVA) showed enhanced osteogenic activity compared to the control. At 4 weeks, the new bone extended into the central defect region, and by 8 weeks, further bone ingrowth led to improved defect bridging and steady integration with the host tissue.

Group BE6 (BGS-7, 600 °C, PEG) did not show substantial differences from Group BV6, despite the difference in binder type. Group BV8 (BGS-7, 800 °C, PEG) consistently showed the most advanced outcomes. At 4 weeks, substantial bone matrix deposition and improved material–host tissue integration were observed. After 8 weeks, extensive defect closure and near-complete bridging were observed, indicating accelerated osseointegration and superior regenerative performance.

#### 3.2.2. Histological Analysis

**Figure 7:**
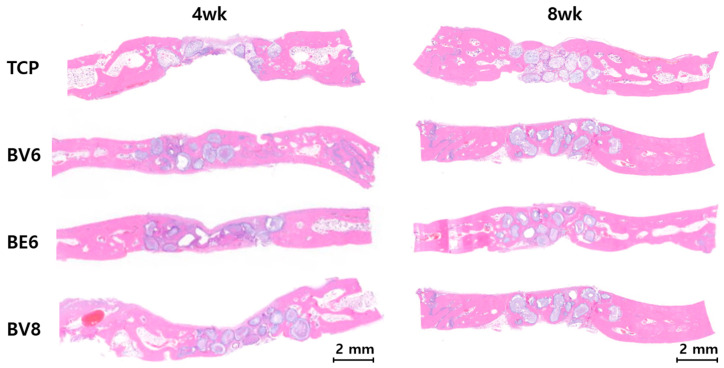
H&E-based histological analysis of calvarial defects at 4 and 8 weeks.

Histological examination using H&E staining further confirmed the differences observed radiographically (Figure 7).

Group TCP displayed minimal cellular infiltration and osteoblast activity at 4 weeks, with fibrous connective tissue predominating. At 8 weeks, this group showed limited bone formation, persistent fibrous tissue, and poor material integration.

Group BV6 showed active bone remodeling after 4 weeks, with osteocytes embedded in the newly formed bone matrix. At 8 weeks, histological examination revealed mature lamellar bone with well-organized collagen fibers and regular osteocyte lacunae, reflecting healthy remodeling.

Group BE6 did not show substantial differences from Group BV6, despite the difference in binder type.

Group BV8 exhibited the most favorable histological outcomes. At 4 weeks, extensive osteoblast activity, collagen deposition, and early mineralization were observed. By 8 weeks, mature bone tissue with well-developed Haversian systems, a dense collagen matrix, and complete mineralization was evident. The bioactive glass material exhibited gradual resorption with concurrent bone replacement.

### 3.3. In Vivo Bone Formation Evaluation in the Rabbit Distal Femur Condyle Defect Model

#### 3.3.1. Radiographic Analysis of Bone Formation

**Figure 8:**
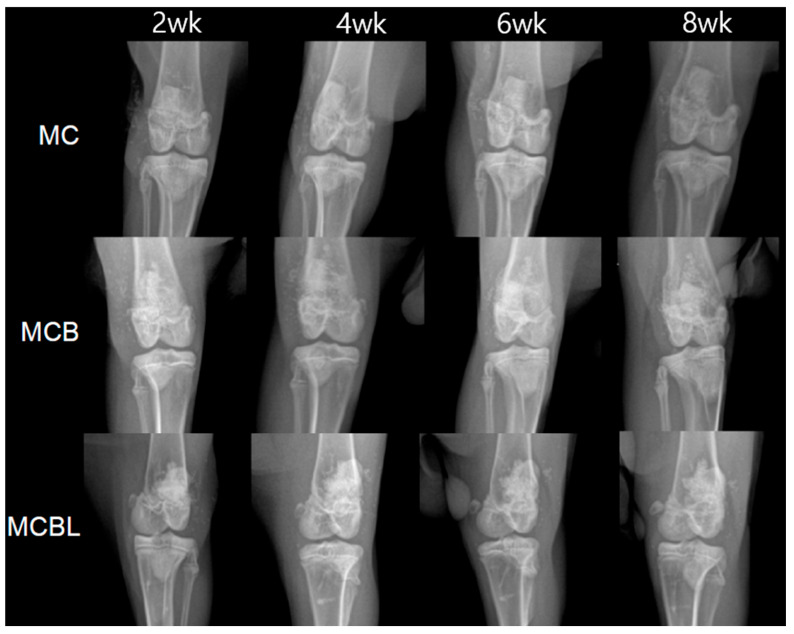
X-ray images of defects at 2, 4, 6, and 8 weeks for each group.

Radiographic evaluations were performed at 2, 4, 6, and 8 weeks post-implantation; the results are presented in Figure 8.

**Group MC:** MC-G gel + BGS-7 macrobeads

Serial radiographic evaluations revealed progressive bone formation throughout the 8-week follow-up period. At 2 weeks post-implantation, initial signs of new bone formation were observed at the defect margins. After 4 weeks, increased radiopacity was evident within the defect site, indicating active mineralization. At 6 weeks, there was substantial bone ingrowth with clear integration between the implant material and surrounding bone tissue. At 8 weeks, near-complete defect filling was observed, with good continuity between the newly formed bone and the host cortical bone. All four subjects showed consistent bone formation patterns with no observable complication.

**Group MCB:** MCB-G gel + BGS-7 macrobeads

The addition of BGS-7 microbeads to the macrobeads formulation resulted in enhanced bone formation compared with that in Group MC. At 2 weeks, radiopaque regions within the defect were more pronounced than that at the previous time point, suggesting accelerated initial bone formation. Progressive bone ingrowth was evident at 4 weeks, with improved defect filling compared with that in the macrobead-only group. After 6 weeks, substantial bone formation was observed with good integration patterns. The 8-week evaluation revealed excellent bone formation with dense mineralized tissue filling most of the defect volume. The combination of macrobeads and microbeads appeared to provide the optimal scaffold architecture for bone ingrowth in all four subjects.

**Group MCBL:** MCBL-G gel + BGS-7 macrobeads

The incorporation of 3% Laponite in the BGS-7 macrobeads/microbeads formulation resulted in distinct bone formation characteristics. Early stages (2–4 weeks) demonstrated initial bone formation comparable to that in Group MCB. However, at 6 weeks, the bone formation pattern appeared more uniform and denser throughout the defect site than that at the previous time point. The 8-week evaluation revealed excellent bone formation with high radiopacity, indicating the presence of mature mineralized tissue. The addition of Laponite enhanced the overall integration and potentially improved the mechanical properties of the newly formed bone. All the subjects showed consistent and robust bone formation.

#### 3.3.2. Micro-CT Analysis at 8 Weeks Post-Implantation

**Figure 9:**
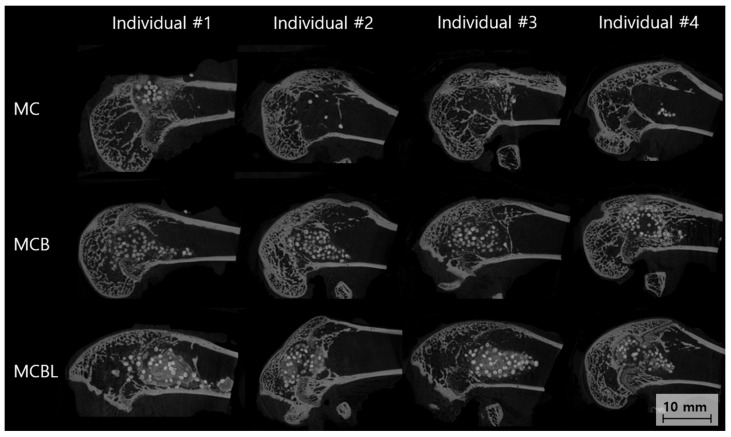
Sagittal Micro-CT images of bone defects at 8 weeks.

At 8 weeks post-implantation, Micro-CT analysis was performed for each defect site; the results are shown in Figure 9.

**Group MC**

The Micro-CT cross-sectional analysis at 8 weeks revealed moderate bone formation within the defect site. Individual #1 showed a partial defect filling with newly formed bone primarily concentrated in the peripheral regions. Individual #2 demonstrated better bone ingrowth with a more uniform distribution throughout the defect volume. Individual #3 exhibited patterns similar to those of #2, with good cortical integration. Individual #4 showed the most complete bone formation among the groups, with dense mineralized tissue filling approximately 70–80% of the original defect volume. The trabecular architecture appeared well-organized, with good connectivity with the surrounding cortical bone.

**Group MCB**

The Micro-CT analysis revealed superior bone formation in Group MCB compared with that in Group MC. Individual #1 showed excellent defect filling, with dense, well-mineralized bone tissue occupying the majority of the defect space. Individual #2 demonstrated outstanding bone formation with near-complete defect obliteration and excellent trabecular architecture. Individual #3 exhibited robust bone ingrowth, with good cortical integration and high bone density throughout the defect. Individual #4 showed complete bone formation with a mature trabecular structure indistinguishable from native bone architecture.

**Group MCBL**

The Micro-CT evaluation showed excellent bone formation with unique characteristics attributed to the incorporation of Laponite. Individual #1 demonstrated dense and uniform bone formation with an exceptionally well-organized trabecular structure. Individual #2 exhibited complete defect filling with high bone density and excellent cortical continuity. Individual #3 exhibited superior bone formation with a mature trabecular architecture and optimal integration with the host bone. Individual #4 demonstrated outstanding bone formation with dense, well-mineralized tissue filling the defects completely. The addition of Laponite appeared to enhance bone quality, resulting in more uniform and denser trabecular patterns than in the other groups.

For quantitative evaluation, the Micro-CT data were analyzed to determine bone volume ratios, including total bone volume (TV), substitute volume (BV), and newly formed bone volume (NV) (Table 4). The NV was significantly higher in all the experimental groups than in the control group (15.7 ± 4.1%, *p* ≤ 0.001). Among the groups, the MCBL group showed the highest NV value (34.1% ± 4.0%), indicating that the incorporation of Laponite remarkably enhanced bone regeneration.

Similarly, the TV was significantly higher in the experimental groups than in the control group (15.9 ± 4.2%, *p* ≤ 0.001), with the MCBL group demonstrating the highest TV (57.7 ± 13.0%). For BV, both MCB (23.1 ± 4.3%) and MCBL (23.5 ± 9.4%) groups had significantly higher values than the MC (8.7 ± 2.2%) and control (0.3 ± 1.2%) groups (*p* ≤ 0.001), confirming the presence and retention of graft materials in the defect area.

**Figure 10:**
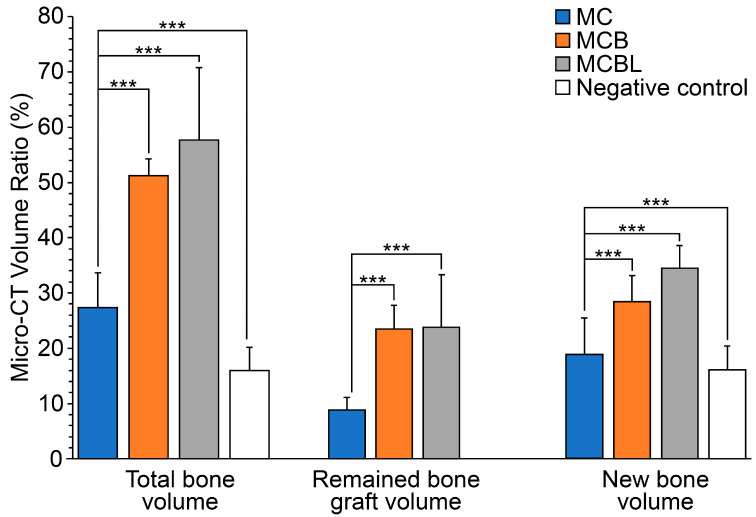
Micro-CT analysis of bone volume ratio: *** *p* < 0.001.

As shown in Figure 10, NV was the highest in the MCBL group, followed by that in the MCB, MC, and control groups. The result further supported the superior osteogenic performance of the MCBL formulation, as quantified using Micro-CT analysis.

#### 3.3.3. Histological Analysis

**Figure 11:**
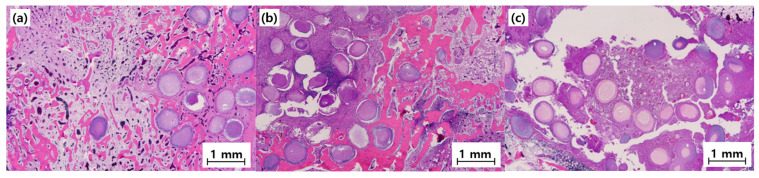
Representative histology images (40×) of bone defects at 8 weeks post-implantation. Scale bar = 1 mm: (**a**) MC, (**b**) MCB, and (**c**) MCBL.

Histological examination using H&E staining further confirmed the differences observed radiographically (Figure 11).

**Group MC**

The histological examination revealed active bone formation, with osteoblast activity evident along the newly formed bone surface. The tissue showed good integration between the BGS-7 macrobeads and surrounding bone tissue. Osteocytes were well distributed within the newly formed bone matrix, indicating healthy bone remodeling. Some residual BGS-7 material was observed, demonstrating gradual resorption and replacement by natural bone tissue. Minimal inflammatory response was observed, confirming good biocompatibility.

**Group MCB**

The histological analysis demonstrated superior bone formation in Group MCB compared with that in Group MC. Dense, mature bone tissue was observed throughout the defect site, with extensive osteoblast and osteocyte populations. The combination of macrobeads and microbeads created an optimal microenvironment for cellular infiltration and bone formation. A well-organized lamellar bone structure was evident, indicating advanced bone maturation. Blood vessel ingrowth was prominent, suggesting excellent vascularization of the newly formed tissues. The BGS-7 materials showed progressive resorption with concurrent bone formation.

**Group MCBL**

The histological evaluation revealed the highest quality of bone formation in all the groups. The newly formed bone exhibited mature lamellar architecture with well-defined Haversian systems. Osteoblast activity was robust, with extensive bone-lining cells observed along the bone surface. The addition of Laponite enhanced cellular activity and promoted a more organized bone structure. Excellent vascularization was evident at the defect site. The tissue architecture closely resembled that of native cortical bone, indicating successful bone regeneration.

#### 3.3.4. Comparative Analysis

A comprehensive evaluation combining the radiographic, Micro-CT, and histological analyses confirmed the superior osteogenic performance of the BGS-7-based formulations. Group MCB (BGS-7 macro + microbeads) and Group MCBL (BGS-7 macro + microbeads + Laponite) demonstrated the most promising results, with the latter showing the highest quality of bone formation. The histological analysis revealed that the addition of Laponite not only enhanced bone formation but also improved bone quality and organizational structure significantly. All animals survived the 8-week study period without any complication. No adverse reaction, sign of infection, inflammatory response, or cytotoxic effect was observed in the experimental groups. The histological analysis confirmed the excellent biocompatibility of all the BGS-7 bioactive glass formulations, with appropriate cellular responses and tissue integration.

## 4. Discussion

This study demonstrated that the BGS-7 bioactive glass significantly outperformed β-tricalcium phosphate (β-TCP) in promoting bone regeneration in rabbit calvarial and femoral condyle defect models. The most important finding is that processing parameters critically influence the osteogenic performance of BGS-7 scaffolds. In addition, formulation strategies combining BGS-7 macrobeads and microbeads with Laponite incorporation achieved near-complete defect closure, with mature trabecular bone histologically indistinguishable from native tissue at 8 weeks.

The superior osteogenic performance of the BGS-7 bioactive glass can be attributed to its unique biodegradation mechanism and bioactive ion release profile. Unlike conventional calcium phosphate ceramics, BGS-7 undergoes controlled dissolution at the defect site, releasing therapeutic concentrations of calcium, phosphate, and silicate ions that create a favorable microenvironment for osteoblast proliferation and differentiation [14,15,16]. The observed enhanced cellular activity and accelerated osseointegration in the BGS-7 groups align with the established mechanism of bioactive glass-mediated bone formation, where silicate ion release stimulates osteoblast gene expression and promotes rapid hydroxyapatite precipitation at the material–tissue interface.

Heat treatment is a critical determinant of BGS-7 performance. Increasing the heat treatment temperature promotes partial crystallization of bioactive phases, which slows degradation but maintains controlled dissolution [19,20,21].

In this study, the 800 °C condition likely optimized the glass network and dissolution kinetics, achieving a balance between scaffold stability and resorption that facilitated bone ingrowth [21]. In contrast, overly rapid degradation at lower temperatures was less favorable. Importantly, BGS-7 specimens treated at both 600 °C and 800 °C showed faster degradation and greater osteogenic potential than β-TCP, a widely used clinical bone graft material, underscoring the translational promise of this composition. These findings align with those of previous studies demonstrating the superior osteogenic potential of bioactive glass over calcium phosphate ceramics [22,24,25]. Our study extends this evidence by systematically examining processing parameters and introducing novel formulation strategies. The poor performance of β-TCP observed here is consistent with reports of uncontrolled degradation kinetics and limited osteoinductive capacity [29,30] and contrasts with occasional favorable reports likely influenced by differences in model design, scaffold architecture, or evaluation timepoints.

Recent work on calcium-incorporated hydrogels has shown that Ca^2+^-mediated crosslinking can enhance the functional stability of hydrogel-based systems in bone applications [31]. The incorporation of Laponite into BGS-7 formulations represents a notable advancement in scaffold design. Here, the dual-scale architecture of macrobeads and microbeads enhanced pore interconnectivity, providing more osteoconductive sites and improving vascular ingrowth while maintaining mechanical stability. Laponite further minimized beads displacement caused by hydrogel swelling and improved osteogenesis through its binding and fixation properties. In addition, the rheological behavior of Laponite ensures stability under normal conditions while permitting mobility under external forces [32,33]. This coupled with its drug-binding capability highlights the dual potential of this hydrogel system containing BGS-7 beads and Laponite for applications in bone regeneration and controlled delivery of therapeutic agents such as antibiotics or growth factors [34].

Clinically, BGS-7 offers several advantages. Optimized formulations could reduce the need for autologous bone grafting in large or complex defects, thereby minimizing donor site morbidity and complications while ensuring consistent outcomes. The addition of Laponite further supports implant retention in irregular geometries and suggests potential for minimally invasive delivery approaches. The demonstrated biocompatibility, osteoconductivity, and controlled degradation position BGS-7 as an attractive candidate for single-stage defect reconstruction, including infected bone defects where multiple surgeries are often required.

From a translational perspective, the optimized processing conditions (800 °C heat treatment) and composite strategy (macrobeads/microbeads with Laponite) provide a clear direction for clinical development. The absence of adverse effects across experimental groups strengthens the rationale for advancing BGS-7 toward clinical trials. The use of two complementary animal models, combined with radiographic, Micro-CT, and histological analyses, provides robust multi-level evidence supporting its efficacy.

Some limitations of this study should be acknowledged. The small sample size (*n* = 2–4 per group) and short-term follow-up (8 weeks) limit generalizability and warrant long-term safety assessment. Healthy animal models may not fully replicate the pathophysiology of infected or compromised bone defects. Additionally, mechanical properties of regenerated bone were not quantitatively assessed, which is essential for load-bearing applications. Viscosity and rheology were not quantified, but all hydrogels showed sufficient injectability and handling for use as carriers. Future studies should address these limitations by using larger samples, longer follow-up, and infected defect models. Dose–response studies are also needed to optimize BGS-7 and Laponite ratios for different defect sizes and anatomical sites.

Further research should explore the molecular mechanisms underlying BGS-7-mediated bone formation, including gene expression and protein-level analyses. Development of injectable formulations and application of 3D printing for patient-specific scaffold fabrication represent additional promising avenues. The most critical next step is to evaluate BGS-7 in infected defect models with antibiotic loading, aiming to achieve both infection control and bone regeneration under clinically relevant conditions. Although infected defect models were beyond the scope of this proof-of-concept study, future work will apply BGS-7 formulations to infected or compromised bone environments to more closely reflect clinical conditions.

## 5. Conclusions

This study establishes BGS-7 bioactive glass as a superior bone graft substitute compared to conventional calcium phosphate ceramics, with optimized processing parameters and novel formulation strategies significantly enhancing bone regeneration outcomes. The demonstrated biocompatibility, controlled degradation, and exceptional osteogenic performance position BGS-7 bioactive glass as a promising tool for addressing the critical clinical need for effective bone defect treatment. These foundational results provide essential preclinical evidence supporting the advancement of BGS-7 bioactive glass toward clinical trials and eventual application in the challenging management of infected bone defects.

## Figures and Tables

**Table 1:** Compositions and fabrication conditions of macrobeads.

Sample	Material	Spherical- Forming Agent	Cross-Linking Agent	Binder	Heat-Treatment (°C)	Bead Size (μm)
TCP	β-TCP	Sodium alginate	CaCl_2_ Solution	PVA	1350	650–800
BV6	BGS-7			PVA	600	650–800
BE6	BGS-7			PEG	600	650–800
BV8	BGS-7			PVA	800	650–800

β-TCP, beta-tricalcium phosphate; BGS-7, CaO–SiO_2_–P_2_O_5_–B_2_O_3_-based bioactive glass; PVA, polyvinyl alcohol; PEG, polyethylene glycol.

**Table 2:** Compositions of hydrogels.

Sample	HPMC	CMC	Carbopol	BGS-7 Microbead	Laponite
MC-G	✓	✓	✓	-	-
MCB-G	✓	✓	✓	✓	-
MCBL-G	✓	✓	✓	✓	✓

Note: ✓ indicates that the component is included; - indicates that it is not included.

**Table 3:** BET-Specific Surface Area of the Fabricated Macrobeads.

	BV6	BE6	BV8
Specific Surface Area (m^2^/g)	1.79	2.78	1.46

**Table 4:** Micro-CT analysis of bone volume ratio (%).

Group	TV ^1^	BV ^2^	NV ^3^
MC	27.3 ± 6.3%	8.7 ± 2.2%	18.6 ± 6.5%
MCB	51.2 ± 3.1%	23.1 ± 4.3%	28.1 ± 4.6%
MCBL	57.7 ± 13.0%	23.5 ± 9.4%	34.1 ± 4.0%
Control	15.9 ± 4.2%	-	15.9 ± 4.2%
*p*	<0.001	<0.001	<0.001

^1^ TV, total bone volume; ^2^ BV, substitute volume; ^3^ NV, newly formed bone volume.

## Data Availability

The original contributions presented in the study are included in the article/Supplementary Materials. Further inquiries can be directed to the corresponding author.

## Supplementary Materials

The following supporting information can be downloaded at: https://www.mdpi.com/article/10.3390/ma19020309/s1, Table S1: The ARRIVE guidelines 2.0: author checklist.

## Abbreviations

The following abbreviations are used in this manuscript:

BGS-7	CaO–SiO_2_–P_2_O_5_–B_2_O_3_-based bioactive glass
β-TCP	β-tricalcium phosphate
PMMA	Polymethyl methacrylate
PVA	Poly (vinyl alcohol)
PEG	Polyethylene glycol
DI	Deionized
HPMC	Hydroxypropyl methylcellulose
CMC	Carboxymethyl cellulose
SEM	Scanning electron microscopy
XRD	X-ray diffraction
IACUC	Institutional Animal Care and Use Committee
H&E	Hematoxylin and eosin
SD	Standard deviation
TV	Total bone volume
BV	Substitute volume
NV	Newly formed bone volume
